# Comment on: Cardiovascular safety of febuxostat compared to allopurinol for the treatment of gout: A systematic and meta‐analysis

**DOI:** 10.1002/clc.23851

**Published:** 2022-05-30

**Authors:** Jian‐Hao Deng, Jia‐Xing Zhang

**Affiliations:** ^1^ Pharmacy Department Longyan Second Hospital Longyan China; ^2^ Pharmacy Department Guizhou Provincial People's Hospital Guiyang China

To the Editor,

We read with interest the systematic review and meta‐analysis by Gao et al.^1^ published in the May 2021 issue of *Clinical Cardiology. *If febuxostat leads to a higher risk of cardiovascular events as compared to allopurinol are debatable. The authors concluded that febuxostat had a better safety profile compared to allopurinol. However, we also believe that there are three important limitations that should be further discussed.

First, this study was a pooled analysis of nine randomized controlled trials (RCTs) and six observational studies including 257 851 patients. It was unknown why the authors selected RCTs and observational studies simultaneously in the same meta‐analysis. RCT is the preferred type of study when included in the meta‐analysis of intervention.^2^ Compared with RCTs, an observational study is usually at higher risk of selection bias which can weaken the reliability of the conclusion. Moreover, combining RCTs and cohort studies in meta‐analysis as done in this article was inappropriate because different study designs and comparison groups may lead to high methodological variability or heterogeneity between studies.^3^ Gao et al.^1^ documented that febuxostat had a lower risk of urgent coronary revascularization and stroke compared with allopurinol. The outcome was inconsistent in our meta‐analysis including RCTs, but similar to our meta‐analysis included cohort studies (Table 1).

**Table 1:** Meta‐analysis of studies that compared the safety of febuxostat therapy and allopurinol treated patients with gout during follow‐up

Outcome	Study design	No. of trial	*I* ^2 ^​​​​​(%)	RR (95% CI)	*p*
Urgent coronary revascularisation	RCT	3	0	1.18 (0.93, 1.51)	.18
	Cohort	2	58	1.27 (1.00, 1.61)	**.05**
Nonfatal stroke	RCT	3	24	1.15 (0.93, 1.43)	.18
	Cohort	3	0	1.16 (1.04, 1.28)	**.005**
Nonfatal myocardial infarction	RCT	4	0	1.20 (1.00, 1.44)	.06
	Cohort	4	82	0.96 (0.70, 1.32)	.82
Cardiovascular death	RCT	6	56	0.93 (0.57, 1.52)	.76
	Cohort	2	87	1.18 (0.39, 3.55)	.77
Death from any cause	RCT	6	78	1.12 (0.70, 1.80)	.63
	Cohort	4	95	1.04 (0.81, 1.34)	.75

*Note*: Bold values denote statistically significant *p* ≤ .05.

Abbreviations: CI, confidence interval; RCT, randomized controlled trials; RR, relative risk.

Second, a thorough literature search is a very important process in a systematic review and meta‐analysis, which can influence the final results. Unfortunately, two eligible published studies (with a relatively large study sample, *n*1 = 2426 participants and *n*2 = 10 519) were missed by the search in this meta‐analysis.^4^, ^5^

Another probably missing critical point is the quality assessment of the included studies. It is a general principle that the high quality of a meta‐analysis directly relies on the included studies' high quality. Several quality assessment scales for different types of studies, which can enable researchers to spot the possible biases in the included studies, are available for this purpose.

We appreciate the authors' efforts in this study and hope that the points we mentioned above will provide a foundation for further discussion.

## CONFLICT OF INTEREST

The authors declare no conflict of interest.
